# Immune profiling of breast cancer patients: relationship between tumour antigen T cell responses with suppressor and effector leukocyte populations

**DOI:** 10.1186/2051-1426-3-S2-P264

**Published:** 2015-11-04

**Authors:** Nicole Janssen, Graham Pawelec, Lisa Speigl, Sotirios P Fortis, Christoforos Haritos, Sonia A Perez, Constantin N Baxevanis, Jithendra Kini Bailur, Christopher Shipp

**Affiliations:** 1 Tuebingen University Hospital, Tuebingen, Germany; 2 Cancer Immunology and Immunotherapy Center, Saint Savas Cancer Hospital, Athens, Greece

## 

It is becoming increasingly clear that the immune profile of breast cancer patients is informative for patient prognosis. Specifically, immune cells with suppressor or effector function have been shown to be valuable in the prediction of patient course on an individual basis. Our previous study has shown that patients with *in vitro* HER2-reactive peripheral CD8+ T cells, high levels of circulating plasmacytoid dendritic cells (pDCs) but low ratios of Myeloid-Derived Suppressor Cells (MDSCs):pDCs and Regulatory T cells (Tregs):pDCs experience a survival advantage. In the present study we have tested T cell responses to additional tumour associated antigens along with an expanded panel of leukocyte phenotypes including myeloid cells (9 different phenotypes including monocytes, MDSCs, pDCs and monocyte-derived dendritic cells (mDCs)), T cells (5 phenotypes including Tregs) and NK cells in a cohort of 50 prospectively enrolled breast cancer patients with early stage invasive ductal carcinoma. Additionally, we have performed an *in vitro* expansion of T cells responding to peptides derived from the tumour-associated antigens Survivin and MUC-1, as well as HER-2 to validate our previous results, and influenza peptides as a positive biological control. To characterise the type of the T cell response to these antigens, responding CD4+ and CD8+ cells were assessed for IFNg, TNF, IL-2, IL-5, IL-10 and IL-17 production per cell by intracytoplasmic flow cytometry, producing 6-cytokine T cell polyfunctionality data. Thus, this study sought to assess the relationship between the distribution of peripheral blood myeloid cells, T cells and NK cells and the presence or absence of *in vitro* T cell responses to tumour-associated antigens in breast cancer. Ongoing analyses will reveal associations between the levels of different leukocyte populations with T cell responses to tumour-associated antigens. Subsequent clinical follow-up will prospectively reveal associations of these factors with patient clinical course.

